# Emerging vancomycin-non susceptible coagulase negative Staphylococci associated with skin and soft tissue infections

**DOI:** 10.1186/s12941-022-00516-4

**Published:** 2022-07-01

**Authors:** Paul A. Akinduti, Yemisi Dorcas Obafemi, Harriet Ugboko, Maged El-Ashker, Olayemi Akinnola, Chioma Jane Agunsoye, Abiola Oladotun, Bruno S. J. Phiri, Solomon U. Oranusi

**Affiliations:** 1 Microbiology Unit, Department of Biological Sciences, Covenant University, Km 10, Idi-Iroko Road, Ota, Nigeria; 2 Department of Internal Medicine and Infectious Diseases, Faculty of Veterinary Medicine, Mansoura University, Mansoura, 35516 Egypt; 3 Laboratory Unit, Covenant University Health Centre, Ota, Nigeria; 4 Department of Microbiology, Federal University of Agriculture, Abeokuta, Nigeria; 5 Central Veterinary Research Institute (CVRI), Lusaka, Zambia

**Keywords:** Coagulase-negative staphylococci, Antibiotic, Communities, Resistance, Vancomycin

## Abstract

**Backgrounds:**

Observable emergence of Vancomycin-Non susceptible Coagulase-negative Staphylococci (VNS-CoNS) associated with skin and soft tissue infections spreading among the urban and rural populace is gradually intensifying severe complications. The isolated VNS-CoNS were evaluated with Matrix-assisted Laser Desorption/ionization Time of Flight Mass Spectrometry (MALDI ToF MS) for species characterization and pan-antimicrobial resistance pattern.

**Methods:**

Out of 256 clinical samples collected including pus, abscess, ear swabs, eye swabs, and aspirates, 91 CoNS isolates were biotyped and further characterized with MALDI-TOF MS. Staphylococci marker genes, Vancomycin susceptibility, and biofilm assays were performed.

**Results:**

Of 91 CoNS isolates, *S.cohnii* (2.3%), *S.condimentii* (3.4%), *S. saprophyticus* (6.7%), *and S.scuri* (21.1%) were characterized with MALDI-TOF with significant detection rate (99.4%; CI 95, 0.775–0.997, positive predictive values, 90.2%) compared to lower biotyping detection rate (p = 0.001). Hemolytic VNS-CoNS lacked *nuc, pvl* and *spa* genes from wound, ear, and aspirates of more 0.83 MARI clustered into a separate phylo-diverse group and were widely distributed in urban and peri-urban locations. MALDI TOF–MS yielded a high discriminatory potential of AUC-ROC score of 0.963 with true-positivity prediction. VNS-CoNS of MIC ≥ 16 µg/mL were observed among all the ages with significant resistance at 25th and 75th quartiles. More than 10.5% of CoNS expressed multi-antibiotic resistance with more than 8 µg/mL vancomycin cut-off values (p < 0.05).

**Conclusion:**

Antibiotic resistant CoNS should be considered significant pathogens rather than contaminant. Biofilm producing VNS-*S. sciuri* and *S. condimentii* are potential strains with high pathological tropism for skin, soft tissues and wound infections, and these strains require urgent surveillance in peri-urban and rural communities.

**Supplementary Information:**

The online version contains supplementary material available at 10.1186/s12941-022-00516-4.

## Introduction

Coagulase-negative staphylococci (CoNS) are now gaining significant importance with much emphasis on the vancomycin-non susceptible Coagulase-negative staphylococci (VNS-CoNS) that were observed to be substantially associated with colonization of the skin, soft tissue, and mucous membranes in different clinical infections [1]. These strains were implicated in severe wound, abscess, skin and soft tissue related infections [2], and it is now gradually emerging to intensify severe complications. The persistence and high tropism of CoNS (particularly *Staphylococcus sciuri* and *S. condimentii* strains), predisposed the populace to unwanted morbidities [3]. Previously, these CoNS were referred to as non-pathogenic commensals, but malnutrition, genetic re-assortment via human and animal interaction [4, 5], and misuse of antimicrobial agents were key driven factors that propel their virulence and resistance to commonly used antibiotics [6].

Vancomycin was an earlier known antibiotic as the first-line drug for staphylococci infection, but the increasing rate of CoNS resistance to this antibiotic is becoming a serious concern in several therapeutics approaches [7]. However, VNS-CoNS are now considered important strains as recent observations have suggested that the prolonged infections with these strains among the hospitalized patients with indwelling devices and in immunocompromised patients [8] were complicated by the expression of multidrug resistance with a resultant treatment failure [3, 9].

Skin and soft tissue infections associated with CoNS are now considered a major pathological issue. The increasing prevalence of *S. saprophyticus *and *S. sciuri* were usually identified as contaminants in infectious disease diagnosis, particularly in low-income settings [10]. Furthermore, poor detection of VNS-CoNS kept enhancing its prevalence and dissemination [11], particularly in low-income countries. The paucity of data on these strains prevalence in peri-urban and rural communities of southwest Nigeria increases the morbidity rate, therapeutic failure, and hospital-acquired infections [12]. Usually, these strains are considered non-significant contaminants but are now emerging and becoming prevalent with very low-level susceptibility to vancomycin, with increasing association with nosocomial infections. The hydrophobicity phenomenon observed among these strains plays a major role in biofilm production and tissue adherence, facilitating infectivity potential and enhancing antimicrobial resistance [13]. The VNS-CoNS strains are now increasing their spread and intensity among the populace. This study aims to characterize the emerging VNS-CoNS strains associated with skin and soft tissue infections and further analyse the strains antimicrobial resistance pattern.

## Methods

### Bacteria strains

Total of 256 clinical samples, including pus (n = 58), abscess (n = 84), ear swabs (n = 62), eye swabs (n = 18), aspirates (n = 34) were collected from out- and in-patients attending the Federal Medical Centre in Abeokuta, Nigeria between June 2018 and March 2019. Inadequate patients’ bio-data is a limitation to have complete demographic status, but they majorly dwell in urban and peri-urban communities in southwest Nigeria. The selection of patients with staphylococcal infection was based on the assessment of the physicians and the Ethical approval for the study was obtained (FMCA/470/HREC/09/2017). Suspected staphylococci colonies from 7% sheep blood agar culture, were phenotypically characterized on Baird-Parker medium, Mannitol salt agar (MSA) and MSA supplemented with Cefoxitin (4 µl/g) as previously described [14]. Typical colonies were further tested for hemolytic activity and coagulase production [15] and further biotyped with Analytical Profile Index (API).

### Matrix-assisted laser desorption/ionization time of flight mass spectrometry (MALDI-TOF MS) analysis

Identified CoNS strains were selected for species confirmation using MALDI-TOF MS [16]. Each strain was sub-cultured on sheep blood agar and incubated overnight at 37 °C to obtain a single pure colony for MALDI-TOF MS analysis. Briefly, a single pure colony of each strain was spotted on a ground steel MALDI target plate in duplicate and allowed to dry. To each spot, 1 μL of the matrix (Bruker Daltonik GmbH, Bremen, Germany), containing a saturated solution of α-cyano-4-hydroxycinnamic acid in 50% acetonitrile (Sigma-Aldrich) and 2.5% trifluoro-acetic acid (Sigma-Aldrich) was added to the spot and was allowed to dry. Thereafter, the spotted plate was placed for analysis in Microflex LT MALDI-TOF MS instrument (Bruker Daltonik GmbH) with the following parameter settings; Ion Source (IS) 1 20 kV, IS2 18.5 kV, lens 8.5 kV, pulsed ion extraction 250 nucleotides with no gating. Spectra calibration and the measurements were carried out for species identification with reference strains, and the highest score for each species was selected according to the manufacturer’s instructions.

### Strain genotyping

Multiplex real-time PCR was adopted for the simultaneous detection of staphylococci marker genes; *tuf* (*Staphylococcus* spp.), *nuc* (*S. aureus), mecA* (methicillin resistance) and *pvl* (Panton-Valentine leukocidin) [17] using the primer sets shown in Additional file 2: Table S1. Briefly, each reaction volume of 20 µl contained 0.8 µl of 10uM primers of tuf-P1, tuf-P2, nuc-P1, nuc-p2, mecA-P1,mecA-P2, pvltaqF and pvltaqR. Probe analyte of 0.2 µl of 10uM each of *nuc, mecA*, and pvlTAQT, DNA template (1 µl) and water (18 µl) were also added. The amplification cycle consisted of an initial denaturation step at 95 °C for 15 min, followed by 30 cycles of denaturation at 95 °C for 15 s, annealing at 60 °C for 60 s programmed and extension 72 °C for 60 s and final extension 72 °C for 5 min. Data evaluation was done with consideration of the negative and positive controls.

### Antibiogram

Susceptibility of the strains to different antimicrobial agents, including gentamicin (GEN), kanamycin (KAN), streptomycin (STR), chloramphenicol (CHL), ciprofloxacin (CIP), tetracycline (TET), clindamycin (CLI), erythromycin (ERY), mupirocin (MUP), linezolid (LZD), vancomycin (VAN), penicillin (PEN), fusidic acid (FUS), cefoxitin (FOX), trimethoprim (TMP), sulfamethoxazole (SMX), vancomycin (VAN), amoxicillin (AMX), ofloxacin (OFX), ceftazidime (CAZ), cefuroxime (CFX), rifampicin (RIF), tiamulin (TIA), and Quinupristin-dalfopristin (SYN) were evaluated for minimum inhibitory concentration (MIC) at dilution ranges of 0.5–128 µg*/*mL using semi-automated broth micro-dilution assay in a commercially prepared microtiter plate [18]. The MICs were interpreted according to the epidemiological cut-off values (ECOFFs) described by the European Committee for antimicrobial susceptibility guidelines (www.eucast.org) [19]. The multi-antibiotic resistance index (MARI) of each strain was also determined [20, 21]. Commonly used antibiotics including TET, CAZ, CIP, GENT, AMX, CFX, OFX, SMX, ERY, FOX; were assayed for susceptibility using disc diffusion method [22] on Mueller–Hinton agar plates. Zones of inhibitions were measured and interpreted according to CLSI guidelines [23].

### Biofilm assay

Seventeen CoNS strains showing MARI of more than 0.2 were selected for biofilm assay, which was performed in microtiter plates [24], following the growth of the strains in tryptic soy broth. Pellet of the bacteria was separated and further diluted to standard suspension of 0.5McFarland turbidity in fresh tryptic soy broth and 200 µl was dispensed into the micro-titer plate and incubated at 37  C for 24 h. After incubation, cells were aspirated away from each well and washed with sterile phosphate-buffered saline (PBS). The plates were dried, and 200 μL of 0.2% aqueous Crystal violet solution was added to each well, and the plates were allowed to stand for 15 min. All the wells were washed again with PBS and 50 µl graded ethanol was added for colouration, which was interpreted for biofilm production according to the criteria described by Stepanovic et al.  [25].

### Vancomycin susceptibility

Vancomycin MICs were graded to evaluate the strain susceptibility using the broth microdilution methods [26]. Briefly, ten serial dilutions of vancomycin (Sigma-Aldrich), were prepared at concentrations of 32 to 0.06 μg/mL and inoculated with standardized bacterial suspension of 0.5McFarlan turbidity. Following the incubation at 37 °C for 24 h, the MIC was determined and interpreted following the CLSI (2018) guidelines [23]. Susceptibility to Vancomycin was classified as Vancomycin susceptible strains (VS-CoNS; MIC ≤ 4 µg/mL); Vancomycin intermediate-CoNS (VI-CoNS; MIC 8–16 µg/mL) and Vancomycin non-susceptible-CoNS (VNS-CoNS; MIC > 16 µg/mL) according to CLSI [23, 27].

### Data analysis

The antibiotic resistance relatedness of the VNS-CoNS isolates were analysed using DendroUPGMA construction utility program to extrapolate the dendrogram from a set of variables based on their respective resistance pattern and MARI. At various distance matrices, similar matrices and transformed similar coefficients were calculated into distances making a clustering with the Unweighted Pair Group Method and Arithmetic mean (UPGMA) algorithm [28]. The significance level of the identified VNS-CoNS strains was determined with Chi-square, and comparative evaluation of the detection rate of biochemical typing and MALDI-TOF analytic methods was performed with pair T-test, taking p < 0.05. Evaluation of MALDI-TOF discrimination of CoNS and VNS-CoNS was performed with ROC curve that provides optimal cut-off value reliability at the area under the curve (AUC) closer to 1 and unreliable at 0.50 or less. Age was considered as a variant for risk factor for the Vancomycin intermediate and VNS-CoNS colonization due to various skin and soft tissue infections that impact on adolescents and young adults quality of life. This evaluation was determined with Mann–Whitney U test, taking level of significant difference at p < 0.05. The antibiotic resistance pattern of the CoNS isolates was estimated with Chi-square for comparisons of resistance proportions to various antibiotics (p < 0.05).

## Results

### Comparative CoNS biotypes detection, biofilm, and resistance pattern

**Fig. 1:**
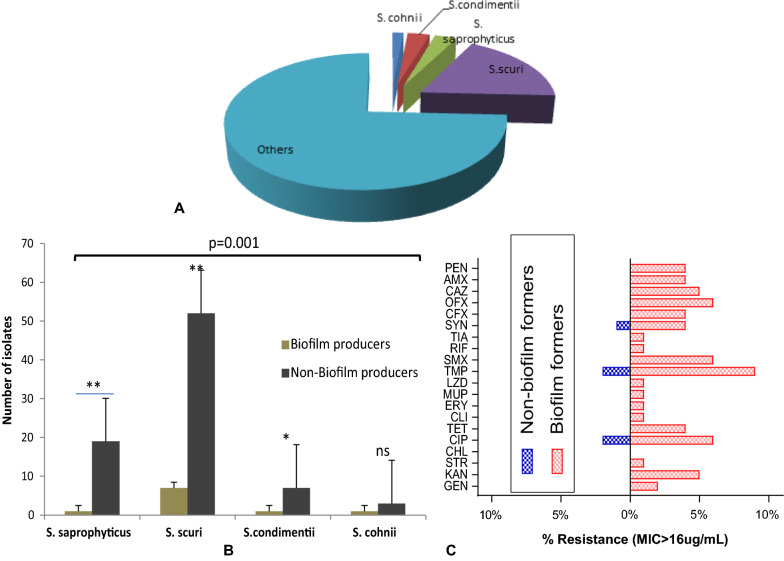
**A** Distribution of MALDI ToF characterized CoNS biotypes obtained from various extra-intestinal sources. **B** Comparative level of biofilm production among the strains of the isolated CoNS. **C** Overall comparison of resistance pattern of CoNS (key: **p = 0.01; *p < 0.05)

Detection of CoNS strains was significantly higher using MALDI-TOF (99.4%; CI 95, 0.775–0.997) with better sensitivity (84.8%), specificity (100%), and positive predictive values (90.2), compared to biotyping analytical detection (75.2%; CI 95, 0.369–0.717) (Table 1). MALDI-TOF MS provided higher accuracy for detection of VNS-CoNS strains (*S. cohnii*, *S. condimentii*, *S. scuri* and *S.saprophyticus*) with spectra signature peak characterizing the strains as shown by the Biotyper data processing (Additional file 1: Figure S1). Out of 91 CoNS isolates, *S. cohnii* (2.3%), *S.condimentii* (3.4%), *S. saprophyticus* (6.7%), *S. scuri* (21.1%) and others (non-staphylococci strains) (66.5%) were identified (Fig. 1A). Significant number of biofilm producing *S. scuri* (7/59) was observed compared to *S. cohnii* (1/4), *S. condimentii* (1/8) and *S.saprophyticus* (1/20) biofilm producers (Fig. 1B). All the biofilm formers showed resistance to all the antibiotics except CHL compared to non-biofilm formers that resisted only CIP, TMP and SYN (Fig. 1C).

**Table 1:** Detection accuracy of MALDI TOF compared to Biotyping analysis

Strains	Strain detection rate (%)	Sensitivity	Specificity	PPV	95% CI	p value
MALDI ToF	99.4	84.8	100.0	90.2	0.775–0.997	0.001
Biotyping	75.2	48.6	45.2	22.2	0.369–0.717	

PPV: Positive predictive value

### Dendrogram analysis of VNS-CoNS

**Fig. 2:**
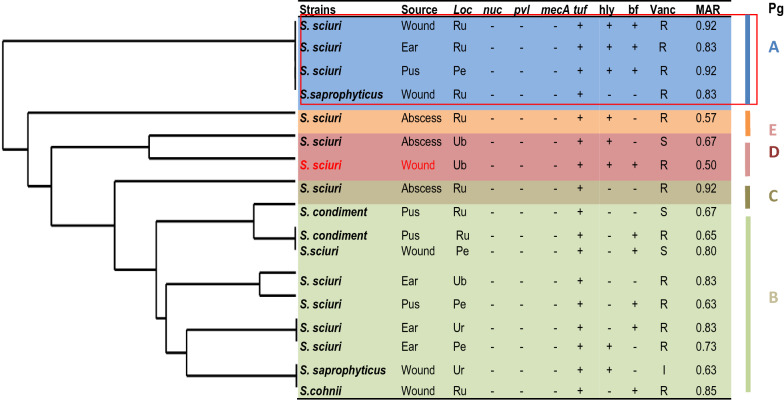
Dendrogram of characterized vancomycin non-susceptible CoNS clustering related multi-antibiotic resistant phenotypes from different locations (Ru, Rural; Pe, Peri-urban; Ur, Urban; hly, haemolysin; bf, biofilm; vanc, vancomycin; MARI, multi-antibiotic resistance; Pg,Phylo-group; R, Resistance; S, Susceptible; I,Intermediate)

Vancomycin non-susceptible *S. sciuri* (wound, ear and pus) and *S. saprophyticus* (wound) with MARI > 0.83 clustered into phlyo-group A having related multi-antibiotic resistance pattern widely distributed in urban and peri-urban locations. Mixed vancomycin non-susceptible *S. condimentii*, *S. sciuri* and *S. saprophyticus* with lower MARI were grouped in phylo-group B while only one strain of vancomycin non-susceptible *S. scuiri* with 0.92 MARI singly clustered to group C (Fig. 2). *S. sciuri* isolated from abscess clustered to group E revealed a lower MARI compared to *S. sciuri* (wound) belonging to group C. Other CoNS from other sources grouped to B also showed a high resistance pattern to vancomycin but lack *nuc, pvl* and *mecA* genes and expressed *tuf* gene.

### Antibiotic resistance patterns of CoNS strains and evaluation of MALDI-TOF MS detection accuracy with ROC

**Fig. 3:**
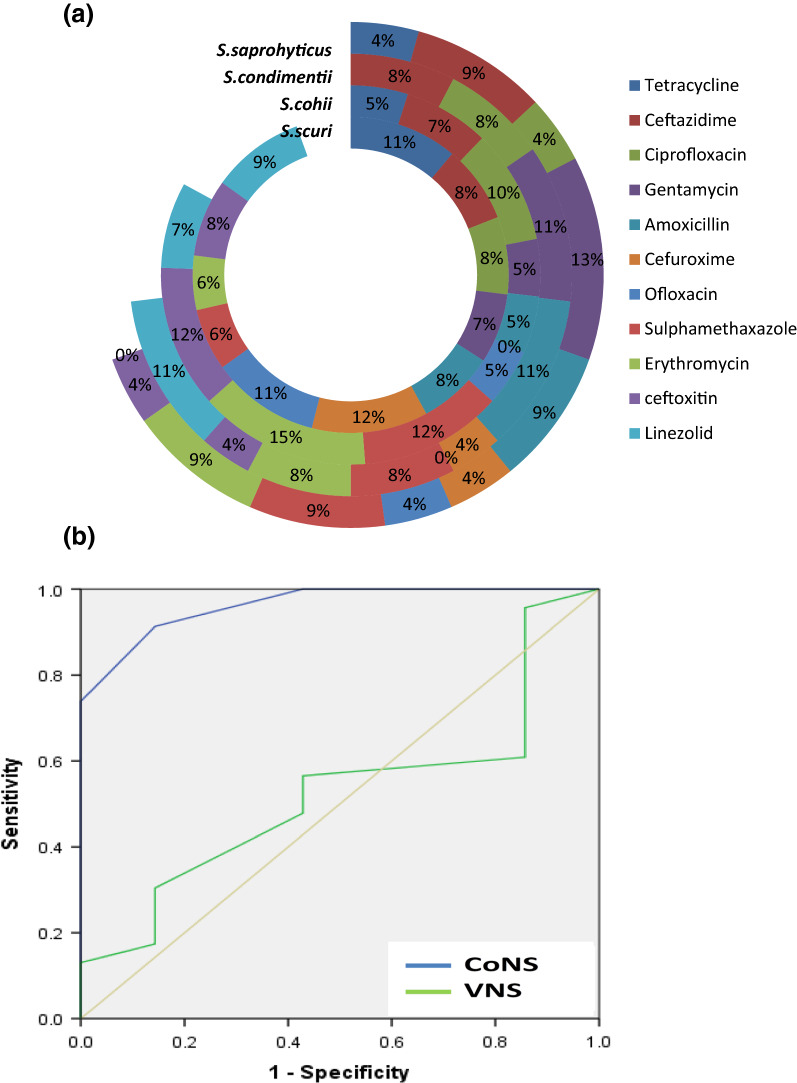
**a** Doughnut chart of the overall resistance of isolated CoNS strains to various antibiotics showing multi-antibiotic resistance pattern. **b** Receiver operating characteristic (ROC) curves for the detection accuracy of the MALDI ToF characterized CoNS strains (AUC = 0.963) and VNS-CoNS isolates with MIC > 16ug/mL (AUC = 0.525)

More than 10.5% of all identified CoNS species showed significant resistance to tetracycline (TET), aminoglycoside (GEN and STR), fluoroquinolone (CIP and ofloxacin), cephalosporin (FOX and ceftazidime) and penicillin (Amoxycillin), linezolid, sulphamethoxazole and erythromycin (p = 0.001; Fig. 3a). ROC was applied to determine the level of accuracy (sensitivity and specificity) of detected CoNS and VNS strains based on the generated area under the curve (AUC). Higher AUC (0.963) for MALDI-TOF MS characterization of CoNS strains could predict a higher level of true-positive CoNS compared to the lower classification of VNS strains (AUC = 0.525) (low sensitivity and specificity rates, Fig. 3b).

### Vancomycin susceptibility: vancomycin susceptibility

**Fig. 4:**
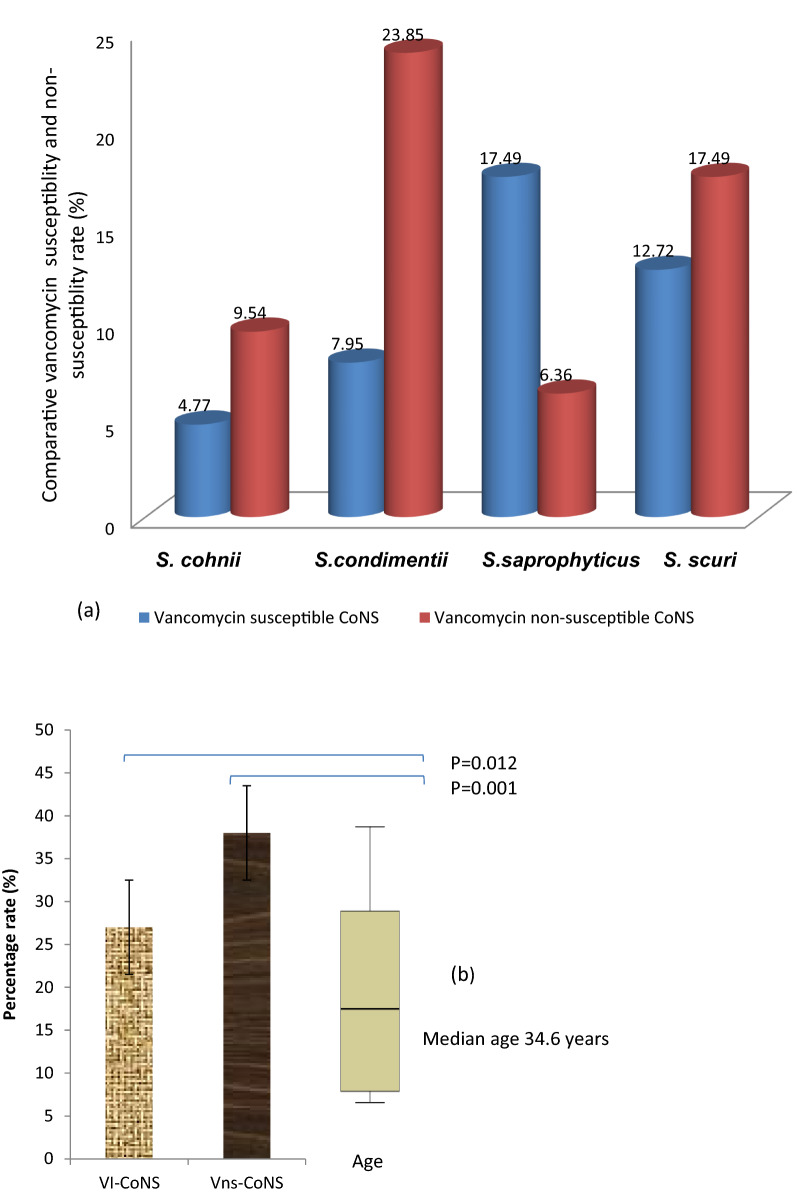
**a** Comparative susceptibility rate of identified CoNS strains to vancomycin. **b** Age variation as a risk factor for the Vancomycin intermediate (VI-CoNS) and Vancomycin non-susceptible (Vns-CoNS)

High significant rate of vancomycin non-susceptible *S. condimentii* (23.85%), *S. cohnii* (9.54%) and *S. saprophyticus* (6.36%) were recorded (Fig. 4a).

(Fig. 4a). Figure 4b also show the overall percentage of VNS-CoNS and VI-CoNS found in the study. Identified Vancomycin non-susceptible CoNS with the MIC > 16 µg/mL were distributed among all the ages (median ≈ 34.6 years) with significant resistance at 25th and 75th quartiles (p = 0.001) compared to intermediate vancomycin susceptibility rate (MIC < 8ug/mL; p = 0.012) (Fig. 4b).

## Discussion

The present study has shown *S. sciuri* and a few other CoNS strains to be rapidly emerging strains associated with skin and soft-tissue infections. However, there are scanty reports on this infection in Nigeria and other African countries [29]. Low immunity due to poor nutrition, unhygienic environment, and strain-specific colonization is a major factor that could likely enhance invasion, persistence, and aggression of CoNS infectivity among the studied population [30]. The use of MALDI-TOF MS for characterization of CoNS further enhances the rapid and precise detection required for robust and accurate diagnosis of CoNS skin infections. Its timeliness and high specificity could aid appropriate disregard for false-positive CoNS, leading to unnecessary antimicrobial use and reduction in skin and soft tissue morbidity [31]. MALDI-TOF MS provided high-throughput detection with greater specificity and predictive power to improve CoNS characterization compared to low detection observed in the use of the biochemical method (biotyping) that relies primarily on culture-based methodologies.

Antibiotic resistance relatedness of hemolytic and biofilm-producing VNS-*S.sciuri* (from wound, ear, and pus) and VNS-*S.saprophiticus* (wound) with high MARI indicate dissemination of phylo-diverse strains. This study identifies some disseminating VNS-CoNS and highlights clusters of strains characterized with biofilm production that would greatly facilitate and intensify adherence, colonization, persistence, and skin tissue tropism. Production of biofilm allows VNS-CoNS adhesion to epithelial surfaces or medical devices, thereby contributing to its colonization, cell proliferation and accumulation in multilayers. In this process, the accumulation of polysaccharide intercellular adhesin (Pia) and adhesive proteins, such as accumulation-associated protein (Aap) and biofilm-associated protein (Bap), will aid protection against the host immune system facilitating virulence. This mechanism also promotes protection from antibiotic attack, further enhanced the development of resistance to antibiotics, which contributes to the survival of these strains and the severity of infection [14, 32, 33].

The observable cluster of VNS-*S sciuri* lacking *pvl*, demonstrating high level heterogenous antibiotic resistance from peri-urban and rural communities, is a pending threat to public health [4, 14]. This further suggests a stemming spread of VNS-CoNS infection of soft tissue, bloodstream and traumatized wounds.

Increasing resistance to the most commonly used antimicrobial drug associated with these strains is a potential factor for therapeutic failure, particularly among the immuno-compromised patients with implants, catheters, severe or septic wounds, traumatized orthopedic and long-term hospitalized elderly patients [34, 35]. The recorded high resistance to various antimicrobial classes (tetracycline, aminoglycoside, fluoroquinolone, cephalosporin, and penicillin), are indication of the acquisition of a large reservoir of resistance determinants via mobile genetic elements, as a result of human–human (hospital-acquired) or human-animal interactions (zoonotic) [36].

Despite MALDI-TOF MS characterization of the emerging VNS-CoNS with improved detection capability, its discriminatory evaluation revealed low true-positive VNS-CoNS detection (AUC = 0.525). Structural mutation of the VNS-CoNS cell wall could give mass spectra of multiple signals of peaks characterized by low m/z values, poor intensities, and low signal-to-noise ratios. Also, ROC provides a reasonable and accurate differentiation of MALDI-TOF AUC level needed for strain typing that could facilitate CoNS infection diagnosis [37, 38]. Application of the ROC algorithm could be considered a rapid, robust, and easy-to-use method for MALDI-TOF discrimination of CoNS types. This technique provides excellent turn-around time for detection and early diagnosis of CoNS infection, particularly vancomycin non-susceptible strains [39]. Another envisaged limitation for VNS-CoNS detection is the selection of suitable filters, high-dimensional classification, computational programming for a relevant subset characterization and classification problems with a large set of diverse specie-features [40].

As for the high rate of VNS-CoNS and VI-CoNS among the subjects of median age 36.5 years, possible misuse of vancomycin drug or its derivatives for skin abrasion, wound or abscess may be inevitable. This would lead to a steady rise in selective pressure for VNS-CoNS, particularly among the adolescent and adult age groups [41]. Vulnerability of young people to staphylococci infection emanates from poor skin hygiene, unguided application of topical antibiotics and skin cosmetics, disposing them to various dermatologic infections [42].

The increasing proportion of VNS-CoNS could provide predictions for unachievable public health antimicrobial stewardship and ineffective antibiotic therapies associated with increased skin and tissue morbidity, mostly among the vulnerable patient groups [43]. VNS-CoNS distribution among all the ages with up to 75th quartile resistance rating calls for immediate surveillance and more clinical attention. Although, inhibitory or cidal activities of related glycopeptides (such as teicoplanin) which is not commonly prescribed, could be impaired via various resistance mechanism. Resistance mechanisms associated with VanA operons could induce phenotypic resistance, resulting in heterogeneous VNS-CoNS identified in wounds and skin aspirates [44]. Mutational changes to cell wall integrity and regulatory genes associated with RNA synthesis, cell transport, and intracellular protein-binding [45], are possible genetic selections that facilitate sequential resistance to vancomycin. These VNS-CoNS are potential emerging threats to therapeutic formulation needed for the treatment of skin and soft-tissue infections. There is a need to prioritize detection, antibiogram, and prevention of these strains, particularly in hospital settings in low-income countries.

Currently, vancomycin is regarded as the best choice for treating staphylococci infection [46], but the prevalence of pan-drug resistance VNS-CoNS would continue to overwhelm its available treatment options and enhance the development of the extended-resistant spectrum, thereby increasing morbidity and occasional mortality, particularly in bloodstream infections.

## Conclusion

Antibiotic-resistant CoNS should be considered significant pathogens rather than mere contaminants and suitable hospital infection prevention and control must be provided. The use of MALDI-TOF MS analytical protocol would enhance diagnosis and aid proper antimicrobial therapy and further improve the definition of VNS-CoNS infections. There is a high tendency for VNS-*S. sciuri* and VR-*S. condimentii* to carry antibiotic-resistant and virulent determinants with the possibility of expressing pathological tropism in the skin, soft tissues, blood-stream, and wound infections. An urgent surveillance would be required to curb unexpected outbreaks in peri-urban and rural communities.

## Supplementary Information

**Additional file 1: Figure S1.** Biotyper data processing of spectra signature characterizing the strains.

**Additional file 2: Tabel S1**. Primers and probes.

## Data Availability

Data sharing is not applicable to this article as no datasets (genomic sequences) were generated or analysed during the current study.
