# Supplementary material for: Emerging vancomycin-non susceptible coagulase negative Staphylococci associated with skin and soft tissue infections
Source: Ann Clin Microbiol Antimicrob. 2022 Jul 1;21:31. doi: 10.1186/s12941-022-00516-4 (PMC9250237; doi:10.1186/s12941-022-00516-4)
Supplement: Supplementary file 1 — Additional file 1: Figure S1. Biotyper data processing of spectra signature characterizing the strains. [file 12941_2022_516_MOESM1_ESM.docx]

**Emerging Vancomycin-Non susceptible Coagulase negative Staphylococci from extra-intestinal infections**

Akinduti Paul, Obafemi Yemisi, Ugboko Harriet, Maged El-Ashker, Akinnola O, Agunsoye Chioma, Oladotun Abiola, Bruno S.J Phiri, Oranusi S.U

Additional file 1: Figure S1; Biotyper data processing of spectra signature characterizing the strains
