# Supplementary material for: Emerging vancomycin-non susceptible coagulase negative Staphylococci associated with skin and soft tissue infections
Source: Ann Clin Microbiol Antimicrob. 2022 Jul 1;21:31. doi: 10.1186/s12941-022-00516-4 (PMC9250237; doi:10.1186/s12941-022-00516-4)
Supplement: Supplementary file 2 — Additional file 2: Tabel S1. Primers and probes. [file 12941_2022_516_MOESM2_ESM.docx]

**Emerging Vancomycin-Non susceptible Coagulase negative Staphylococci from extra-intestinal infections**

Akinduti Paul, Obafemi Yemisi, Ugboko Harriet, Maged El-Ashker, Akinnola O, Agunsoye Chioma, Oladotun Abiola, Bruno S.J Phiri, Oranusi S.U

Additional file 1: Table S1; Primers and probes

| Target gene | Primer name | Primer sequences 5’- 3’ |
| --- | --- | --- |
| *tuf* | Tuf-P1 | AAACAACTGTTACTGGTGTAGAAATG |
|  | Tuf-P2 | AGTACGGAAATAGAATTGTG |
|  | Tuf Probe | TCCGTAAATTATTAGACTACGCTGAAGC |
| *nuc* | Nuc-P1 | GTTGCTTAGTGTTAACTTTAGTTGTA |
|  | Nuc-P2 | AATGTCGCAGGTTCTTTATGTAATTT |
|  | Nuc Probe | AAGTCTAAGTAGCTCAGCAAATGCA |
| *mecA* | MecA-P1 | AAATATTATTAGCTGATTCAGGTTAC |
|  | MecA-P2 | CGTTAATATTGCCATTATTTCTAAT |
|  | MecProbe | CAAGGTGAAATACTGATTAACCCAGTA |
| *pvl* | pvltaqF | AATGAAATGTTTTTAGGCTCAAGACA |
|  | pvltaqR | TGGATAACACTGGCATTTTGTGA |
|  | pvlTAQT Probe | AGCAACTTAAATGCTGGACAAAACTTCTTGGAA |
